# The Effects of the Refugee Crisis on Age Estimation Analysis over the Past 10 Years: A 16-Country Survey

**DOI:** 10.3390/ijerph14060630

**Published:** 2017-06-13

**Authors:** Leanne Sykes, Ahmed Bhayat, Herman Bernitz

**Affiliations:** 1 Departments of Prosthodontics, School of Dentistry, Faculty of Health Sciences, University of Pretoria, Pretoria 0001, South Africa; leanne.sykes@up.ac.za; 2 Department of Community Dentistry, School of Dentistry, Faculty of Health Sciences, University of Pretoria, Pretoria 0002, South Africa; 3 Department of Oral Pathology and Oral Biology, School of Dentistry, Faculty of Health Sciences, University of Pretoria, Pretoria 0002, South Africa; bernitz@iafrica.com

**Keywords:** age estimation, asylum seekers, refugees

## Abstract

Dental age estimation (AE) tests are routinely done on living and deceased persons. There is anecdotal evidence suggesting an increase in age estimations due to the refugee crisis. Our aim is to determine the reasons and methods for performing dental AE tests in both living and deceased individuals. Global trends in AE over the past 10 years were also investigated. A database of all forensic laboratories was obtained and an electronic questionnaire was sent to all of them. The questionnaire was self-developed and included questions on the reasons for performing AE tests, the preferred methods used in living and deceased individuals, and the people/organizations who requested these AE tests. The number of tests performed annually varied between 0 and 500 and the majority were on asylum seekers, refugees, and for adoption cases. Most units used multiple techniques to determine the age among the living, but seldom used more than three techniques for the deceased. The majority of tests were requested by coroners and the legal fraternity. There has been an increase in the number of dental AEs carried out and this has been mostly due to asylum seekers and refugees. The most common techniques for the living were variations of Demirjian’s technique while country specific techniques were used for the deceased.

## 1. Introduction

In South Africa, there is anecdotal evidence to suggest that there has been an increased demand for age estimation (AE) on living subjects. This could be due to the influx of asylum seekers, illegal immigrants, and more juvenile offenders being tried in the courts. There are many methods used for AE in forensic medicine, with the most common involving somatic, sexual, skeletal, and dental maturity [1,2]. Rapidly improving technology has seen an increase in the development of new as well as the refinement of existing techniques [3]. Age estimation is performed in both living and deceased subjects, but is more difficult in the former, where the demand seems to be increasing internationally [4]. This is mostly for criminal proceedings, where age may dictate whether or not a suspect is old enough to be charged with a crime, and for immigrants seeking asylum, which is often only granted on condition that the person is a minor [4].

When deciding on an AE technique, one has to consider the context, costs, time, equipment required, examiner’s qualifications, the stage of preservation of the body (in deceased persons) as well as how accurate, precise, and reliable the diagnosis needs to be [5]. Dental AE is an extrapolation by a probability procedure based on dental growth often in conjunction with radiographs [6]. Therefore, it must be noted sexual, dental, or skeletal age are not necessarily the same in a single individual and that deviation does exist between the biological and chronological ages [6].

Different methods are available for estimating the dental age and these include AlQahtani, Haavikko’s, Demirjian’s, and modified Demirjian’s methods [5,7]. Currently, countries choose a method based on their population and on past experience and there is no consistency in the types of techniques being used across the globe [7].

One of the problems associated with the dental AE methods is the use of radiographs. The taking of radiographs has ethical issues since it is considered an invasive process [6]. As such the taking of radiographs for AE must be guided by ethical principles [8]. Therefore, since 1996, the Royal College of Radiologists reported that radiation should be used only in cases of clinical need and, therefore, for AE analysis, medical indications have to be authorized by an authority order [9].

This study is the first to identify the reasons and tests used to estimate the age in different countries.

The aim was to determine the changing trends in dental AE methods over the past 10 years and to determine the most commonly used methods in both living and deceased individuals.

## 2. Methods

A retrospective record based cross sectional analytical study design was used. A list of all forensic odonto-stomatological units and contact persons was obtained from the International Organization for Forensic Odonto-Stomatology (IOFOS). A cover letter together with an electronic questionnaire was sent to each unit inviting them to participate. If there was no response from the unit, a second and final request was sent to the head of the respective unit. The questionnaire was self-developed since no similar study has been done in the past. It was in the English language and submitted electronically to the contact person at each of the facilities. The questionnaire elicited information related to the reasons for performing AE tests, the type of tests being used, and if the trends have changed over the past 10 years (from 2005 to 2015). Some of the questions included name of country; types of age estimation analysis test routinely used; reasons for performing the AE analysis; and open-ended comments.

Confidentiality was guaranteed, but each unit’s location (country) was recorded in order to compare the different profiles in each country. Those units that did not respond at all, could not respond due to language barriers, or had incomplete responses were excluded from the study. 

The study was reviewed and approved by the Faculty of Health Sciences Research Ethics Committee of the University of Pretoria (ID: 2015/35).

## 3. Results

Out of a total of 29 units, 23 forensic odonto-stomatological units from 16 different countries responded to the questionnaire. These countries included: Australia, Belgium, Brazil, Canada, Chile, Denmark, Finland, France, India, New Zealand, Norway, South Africa, Sri-Lanka, and Sweden. Although all of them reported to carry out AE analysis, there was a wide range in the number of AE tests being performed annually. The reasons for the AE tests included identification of skeletal remains and in mass disasters or for criminal cases. The demand for these tests increased by 9% over the study period. However, the demand for AE in asylum seekers and refugees increased by 26% and 13%, respectively.

Overall, the number of AE tests ranged from 0 to 500 in 2014 with some units having carried out almost 3000 AE tests in the last 10 years. In 2014, most of the AE tests were performed by Sweden (500) and Denmark (400). The reasons for the tests included the identification of skeletal remains, age of asylum seekers, criminal cases, routine work, mass disasters, and fetuses. Sweden cited an increase in AE tests due to asylum seekers, immigrants, and for adoption purposes. Chile reported to perform the third highest number of tests (200), however this was a newly established teaching unit and all were retrospective cases used for educational purposes.

In the 10-year analysis, Norway, Sweden, and Denmark were again amongst the top three (over 1900 each) with Norway being the highest at 3000 cases. Sri Lanka, a newly established unit, was fourth with 523 cases since 2009 and noted an increase in demand for all types, but in particular civil cases for those with no birth certificates.

**Figure 1:**
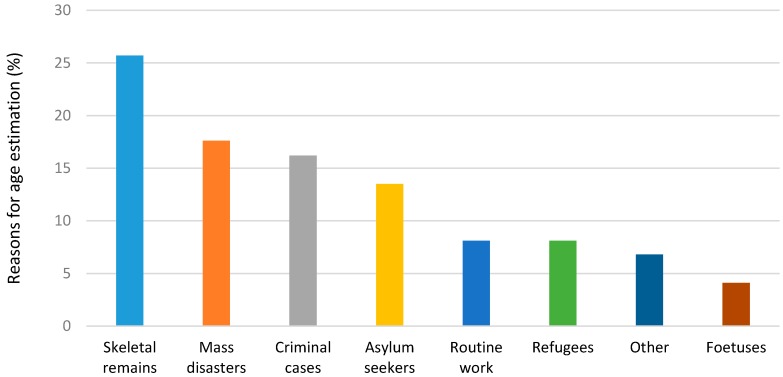
Reasons for age estimation requests (%).

The main reasons for AEs were for identification of skeletal remains (25.7%), mass disasters (17.6%), criminal cases (16.2%), asylum seekers (13.5%), with fetuses being the lowest (4.1%). Cases noted as “other” included lack of birth certificates, adoptions, teaching and learning purposes and teenagers in judicial cases (Figure 1).

**Figure 2:**
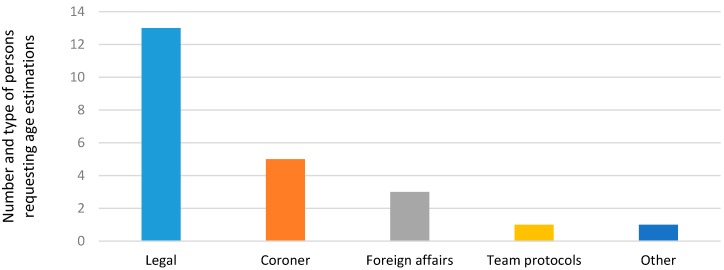
Persons requesting age estimation.

The requests for AE tests were from legal sources such as courts, criminal offices, judges, magistrates, police, and legal medicine departments. Followed by coroners, foreign affairs, team protocols, and other (Figure 2).

**Figure 3:**
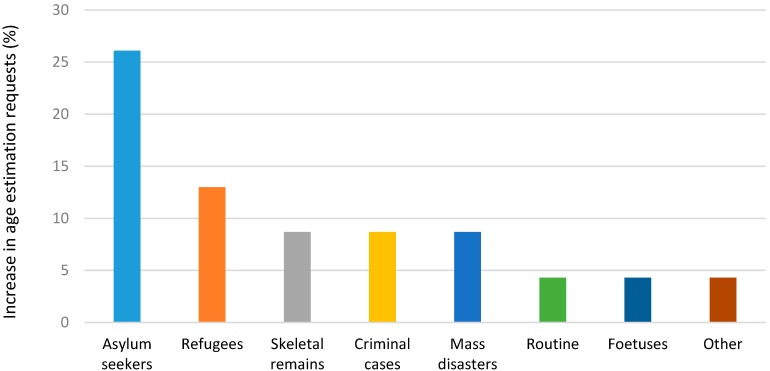
Percentage increase in the number of requests per category (%).

Generally, most units noted an increase in the number of requests for AE tests with the highest among asylum seekers (26%) and refugees (13%) (Figure 3).

**Figure 4:**
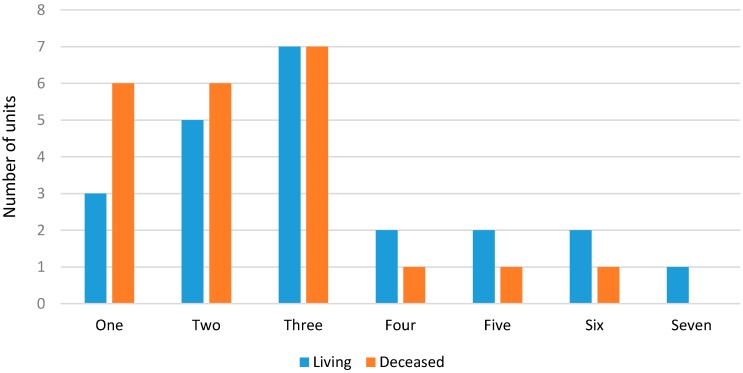
Number of methods used by each unit in living and deceased subjects.

To determine the age among living subjects, most units used three (30%) or two (22%) methods, while amongst deceased subjects, many units used between one (26%), two (26%), or three (30%) different methods (Figure 4).

**Figure 5:**
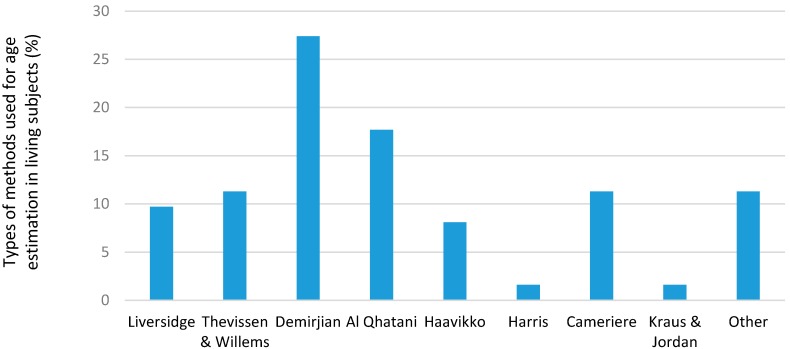
Types of methods used for age estimation in living subjects (%).

**Figure 6:**
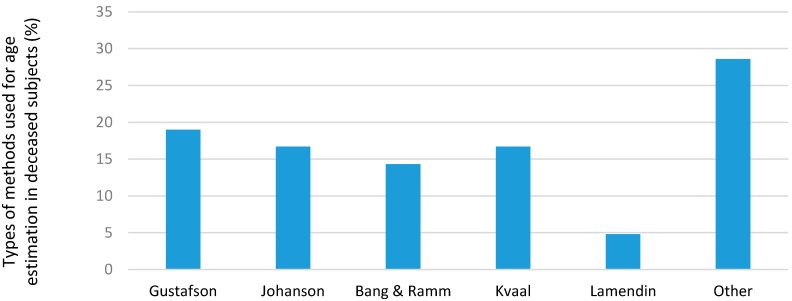
Types of methods used for age estimation in the deceased (%).

Different methods were used to determine the dental age at the different units for living and deceased subjects (Figure 5 and Figure 6). In the living subjects, the most common methods used were the Demerjian and AlQahtani methods; in deceased subjects, the most common methods were the Gustafson, Johansen and Kvaal methods. Many units listed other types of methods but did not specify the name or type used.

## 4. Discussion

Age estimation analysis were being performed across the globe for various reasons. The number of AE tests increased by 26% and 13% for asylum seekers and refugees which indicted the changing trends in AE analysis over the past 10 years.

The reasons for performing AE tests varied as listed below:

- **South Africa**—criminals claiming to be younger in order to receive leniency in judgements against them.
- **Chile**—in sexual abuse and rape cases and in the identification of skeletal remains a major earthquake five years ago.
- **Belgium**—due to an increase in refugees seeking asylum.
- **Sri Lankan**—on suspected Somalian pirates as well as at detention homes for psychiatric patients.
- **India**—for marriage purposes where participants must be older than 18 and 21 years for females and males, respectively. Another reason is to determine the age of athletes participating in various national and international sports events, to prevent older participants taking part in a younger category.
- **Sweden**—for adopted and immigrant children. It assists in the correct placement at schools, as well as for young asylum seekers in order to establish their age.
- **New Zealand**—mass fatality incidents and human identification.

The majority of requests for AE analysis were from legal sources and coroners. When analyzing the results of the different methods used, generally more were used for living subjects than for the deceased. This is because the implications, and thus the need for accuracy, are far more crucial for the former, and many legal systems demand results to be validated by more than one method. This has led to extensive research being carried out on living subjects, and a number of centers of excellence have developed under the direction of renowned authorities such as Dr. Liversidge (Institute of Dentistry, Bart’s and the London School of Medicine and Dentistry, Queen Mary University of London), Prof. Thevissen (Forensic Odontology, Faculty of Medicine, Universiteit Leuven) and Prof. Cameriere (Institute of Forensic Medicine, University of Macerata, Italy). It is also in agreement with other studies that as many techniques as possible should be used for age estimation to improve accuracy and establish maximum reproducibility [10]. The most commonly used method was Demirjian’s, closely followed by Al Qahtani and then Thevissen. This was reflected in the total percentages as well as the percentage of cases. Listed amongst other were Anderson’s attrition scores, T-Takie charts, Schour & Massler, Moorrees, the ABFO system, the “Brazilian” method, and Mincer and Kullman. A point to note was that many sites used variations or derivatives of the Demirjian’s method which could account for its high scores.

Many of the published methods use population specific data tables. For example, Harris and Norje is based on a colored population, Demirjian’s on a French-Canadian group, and Haavikko on a Finnish sample. However, only four centers—India, Brazil, Switzerland, and Australia—mentioned using their own methods and/or population specific data tables. It would have been interesting to inquire from all units if they had similar population specific tables, or merely relied on the general tables, and whether this would make any difference. As one respondent commented “*I wonder how long we are going to be able to use the “different” tables for different races as we are facing more globalization of the entire world with interracial marriages. I guess this will be our next challenge: to prove that human teeth develop in a similar way globally.*” As populations migrate and mingle these racial distinctions may become more blurred. In South Africa, there is already a trend to classify persons as “self-reported whites” etc., but this is based on self-identity and may not be a true reflection of their ancestry. This point may be even more relevant in deceased individuals, where the ethnic origin is often unknown.

Comments based on the tests used for living respondents:

- **New Zealand**—uses the most non-invasive methods wherever possible, and stressed that age estimations should always be given as a range.
- **Sweden**—use a combination of visual and statistical methods, but also examine the person clinically.
- **Belgium**—all methods applicable to the available evidence were used for adults, while in children and sub-adults, they use various methods including those described by Thevissen and Willems.
- **Canada**—use the same three methods for the living and the deceased, namely Thevissen, Demirjian, and AlQahtani.
- **Australia**—recently changed to a modification of Demirjian’s as well as the Taylor & Blenkin method with the Australian age stats pictorial (mentioned above).
- **Switzerland**—units consult four relevant references for different age estimation methods.
- **Brazil**—used their own “Brazilian method” (devised by Nicodemo, Morais, and Medici), as well as Gustafson, with the modification by Olze and Kvaal for living subjects, despite not having done any in recent years.
- **India**—modify or adapt their methods to suit the local Indian populations in terms of regression formulae. Other methods include CRETOT, charts of Ubelaker, Charts of Schour & Massler, Anderson’s attrition scores, T-Takie scores, Moorrees, the ABFO system, and Mincer and Kullman.

In the deceased, the majority of units used one AE method while the remaining units used more than three. The most commonly used methods were Gustafson, Johanson and Kvaal. However, there was a wide range of “other” methods listed that did not appear in the questionnaire, and most units used one of these comments based on the tests used for deceased respondents:

- **Switzerland** use tooth cementum annulation (Wittwer-Backofen).
- **France** use the dental root color card of Collet; and the method of Henri Lamendin.
- **India** reported to modify their methods to suit the local Indian populations. Other methods included Thevissen, Willems, Cameriere, Li and Ji stages of attrition, Solheim, root dentine translucency, and the ABFO method.

This research supports the perception and current literature, citing an increase in demand for age estimation from asylum seekers and refugees. Many of these are unaccompanied minors, seeking protection to escape from persecution, for family unification, economic enhancement, to join other migrant groups, fleeing victim trafficking or smuggling, for medical reasons, or who have run away from home or been abandoned [11]. Accurate age estimation in this group is crucial, as it may have legal implications given that the attainment of the age of majority differs according to the law in each country. It is needed for the “entry” country to decide how to handle these young immigrants, as well as when considering questions relating to criminal activity, forced marriages, sporting eligibility, the right to schooling and education, legal responsibility, and the right to drive and vote [10].

In the sub-adult population, accurate AE is more difficult, as children develop and mature at vastly different rates based on both genetic and environmental influences. Thus, it has been suggested to use a combination of dental and other age estimation methods including radiological investigations of dental and skeletal development [11,12]. Thevissen et al. [13] compared nine different techniques to try establishing which was best for use in the sub-adult population group. They found the method of Moorrees to be the most promising while that of Cameriere fared the worst. This was explained because the latter registers continuous data, and used actual measurement ratios, while the others were based on staging and scoring. They also noted that despite statistical differences, there was no real clinical difference between any of the nine techniques used as long as the chosen method did not compromise the feasibility of correctly registering all the described stages [13]. They also advised that age predictions were drastically improved when cervical vertebrae development was added to the third molar development, and suggested using the Seedat et al. [14] cervical measurement method in combination with their method for third molar development. Another factor to consider in sub-adults is that many of the dental AE methods used in this group are modelled on “retrospectively collected third molar development data, yet an undisputed assessment should be based on a sample from the same origin as the examined individual” [14]. In spite of the fact that much literature has been published showing statistically significant differences in third molar development between different racial groups and genders, studies reported only slight differences in AEs when using a combined country specific data set as opposed to a single growth chart [15]. It is recommended however, to use gender specific tables whenever possible, and that all reports should state the most probable age, give a range of scatter of the reference population, account for observer error, and state which reference study data was used [15,16].

## 5. Limitations

Although the study included 16 countries, it does not represent all AE sites in the world and the results need to be interpreted with caution.

## 6. Conclusions

There was an increase in the number of AE tests that were performed annually over the past 10 years. The majority of them were carried out on asylum seekers, refugees, and adoption cases. Variations of the Demirjian's technique and the AlQahtani method were most commonly used in living subjects while the Gustafson and country specific techniques were commonly used for the deceased. Future studies should focus on collaboration between all forensic units across the globe to allow for sharing of expertise and technology and to help bolster this field internationally.
